# *In situ* injectable chemico-biological cascade-driven antioxidant nanoparticles for periodontitis treatment

**DOI:** 10.1093/rb/rbaf125

**Published:** 2025-12-08

**Authors:** Haoran Ning, Xin Qi, Wa Li, Qing Wu, Xiaochen Liu

**Affiliations:** Shanghai Engineering Research Center of Tooth Restoration and Regeneration & Tongji Research Institute of Stomatology & Department of Endodontics, Shanghai Tongji Stomatological Hospital and Dental School, Tongji University, Shanghai 200072, China; Department of Stomatology, Shanghai Fifth People’s Hospital, Fudan University, Shanghai 200240, China; Department of Stomatology, Shanghai East Hospital, Tongji University School of Medicine, Shanghai 200120, China; Department of Stomatology, Shanghai Fifth People’s Hospital, Fudan University, Shanghai 200240, China; Shanghai Engineering Research Center of Tooth Restoration and Regeneration & Tongji Research Institute of Stomatology & Department of Endodontics, Shanghai Tongji Stomatological Hospital and Dental School, Tongji University, Shanghai 200072, China

**Keywords:** periodontitis, reactive oxygen species elimination, antioxidant nanoparticles, thermosensitive hydrogel, macrophage polarization

## Abstract

Oxidative stress in the periodontal microenvironment intensifies inflammation and accelerates alveolar bone destruction. Consequently, strategies that effectively suppress oxidative stress while promoting osteogenesis are central to the management of periodontitis. Here, we present an *in situ* injectable antioxidant nanoparticle system designed to initiate a sequential chemico-biological cascade, achieving dual therapeutic outcomes of inflammation suppression and bone regeneration. The engineered nanoparticles were fabricated by encapsulating 4-octyl itaconate (4OI) within mesoporous polydopamine nanoparticles [4OI-loaded mesoporous dopamine (MDAI)]. Following cellular uptake, MDAI activates a two-step antioxidant mechanism. First, the mesoporous polydopamine scaffold undergoes ROS-triggered degradation within inflammatory macrophages, directly scavenging excessive ROS. Subsequently, the released 4OI activates the Nrf-2/HO-1 signaling axis, leading to robust antioxidant and cytoprotective effects, as evidenced by the pronounced upregulation of Nrf-2 and modulation of HO-1 activity. This signaling cascade shifts macrophage polarization toward the anti-inflammatory M2 phenotype and suppresses pro-inflammatory cytokines such as tumor necrosis factor alpha and interleukin 6. Transcriptome sequencing further confirmed broad downregulation of inflammatory pathways and associated genes. Moreover, the ROS-scavenging activity of MDAI indirectly enhanced osteoblast differentiation and bone formation. When incorporated into a thermosensitive hydrogel for localized administration, MDAI exhibited prolonged retention and sustained bioactivity within periodontal pockets. In a murine periodontitis model, this formulation effectively reduced inflammatory infiltration, decreased cytokine expression, modulated macrophage polarization and enhanced alveolar bone regeneration. Collectively, these findings establish MDAI-mediated chemico-biological cascade therapy as a potent and integrative platform for treating periodontitis and restoring periodontal tissue homeostasis.

## Graphical abstract

**Figure:**
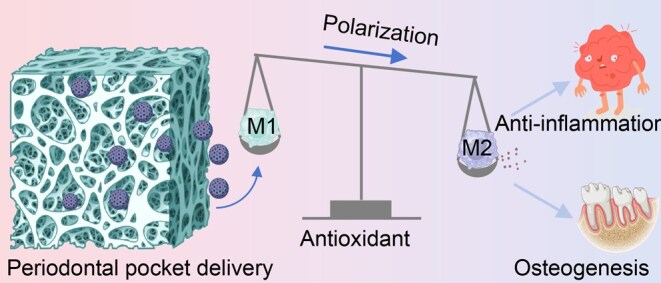

## Introduction

Periodontitis is a widespread chronic inflammatory condition that arises from persistent microbial biofilm accumulation, dysregulated host immune responses and progressive destruction of periodontal tissues, ultimately leading to alveolar bone resorption [1, 2].

Mounting evidence identifies oxidative stress within the periodontal niche as a central pathological trigger that fuels inflammation and tissue degeneration [3, 4]. Overproduction of reactive oxygen species (ROS) not only inflicts direct oxidative injury on resident cells but also activates pro-inflammatory signaling networks such as nuclear factor kappa B (NF-κB), initiating cytokine cascades characterized by elevated tumor necrosis factor-alpha (TNF-α), interleukin 6 (IL-6) and other inflammatory mediators [5, 6]. This sustained oxidative–inflammatory feedback inhibits osteogenic differentiation, stimulates osteoclast activity and accelerates irreversible bone loss [7, 8]. Consequently, restoring redox equilibrium to simultaneously regulate inflammation and promote osteogenesis has emerged as a crucial therapeutic direction for overcoming the limitations of conventional treatments [9, 10].

Existing clinical interventions, including scaling and root planing, primarily target bacterial eradication but fail to modulate the underlying immune dysregulation that perpetuates chronic inflammation [11–13]. Similarly, conventional antioxidants such as N-acetylcysteine and vitamin E exhibit transient efficacy due to short biological half-lives, poor tissue specificity and limited capacity to interrupt inflammatory feedback loops [6, 14]. Their single-mode mechanism of ROS scavenging is insufficient to reprogram the inflammatory microenvironment. Among the immune cells involved, macrophages are recognized as pivotal regulators in the progression and resolution of periodontal inflammation. An imbalance between the pro-inflammatory M1 phenotype and the reparative M2 phenotype determines whether tissue destruction persists or healing occurs [15, 16]. Accordingly, designing therapeutic systems capable of modulating macrophage polarization while restoring the osteogenic milieu represents an urgent need in periodontitis therapy.

Antioxidant nanoparticles, also referred to as ROS-scavenging nanomaterials, have emerged as an advanced class of biomaterials capable of catalytically neutralizing ROS and mitigating oxidative damage in chronic inflammatory diseases [17]. Systems such as Prussian blue-based and manganese-doped nanoparticles exhibit the ability to eliminate superoxide anions, hydroxyl radicals and hydrogen peroxide (H_2_O_2_), thereby suppressing oxidative stress, attenuating inflammation and protecting alveolar bone integrity [18]. Beyond their redox regulation, these nanoparticles exert immunomodulatory effects by promoting macrophage polarization from the M1 to M2 phenotype and enhancing osteoblast differentiation [19, 20]. Their high surface area, porosity and modifiable interfaces allow efficient drug encapsulation, improved bioavailability and controlled release at inflamed sites [21]. Despite these merits, existing antioxidant nanoplatforms remain constrained by monofunctional catalytic activity, which cannot fully resolve persistent inflammation. Likewise, small-molecule immunomodulators such as 4-octyl itaconate (4OI) suffer from inadequate delivery efficiency and poor intracellular retention, limiting their therapeutic potential [22–24]. To address these challenges, multifunctional nanosystems capable of integrating redox regulation, immune modulation and osteogenic stimulation are essential for comprehensive periodontitis therapy.

Sustained and localized drug activity within periodontal pockets is equally vital for long-term disease control. An optimized delivery vehicle that ensures extended retention and controlled drug release can markedly improve therapeutic durability and spatial precision [25, 26] Thermosensitive hydrogels are particularly advantageous for this purpose, as they undergo sol–gel transitions near physiological temperature, enabling *in situ* gelation after administration [27, 28]. This phase transition prolongs drug retention at the target site, thereby enhancing therapeutic efficacy and reducing systemic side effects. In its liquid state at lower temperatures, the hydrogel allows facile drug incorporation and convenient administration. Upon gelation at physiological temperature, it provides a stable localized depot that optimizes site-specific drug release, making it particularly advantageous for treating localized inflammatory conditions such as periodontitis. Moreover, its biocompatible sol–gel transition temperature supports patient comfort and compliance during application [29, 30].

**Figure 1.**
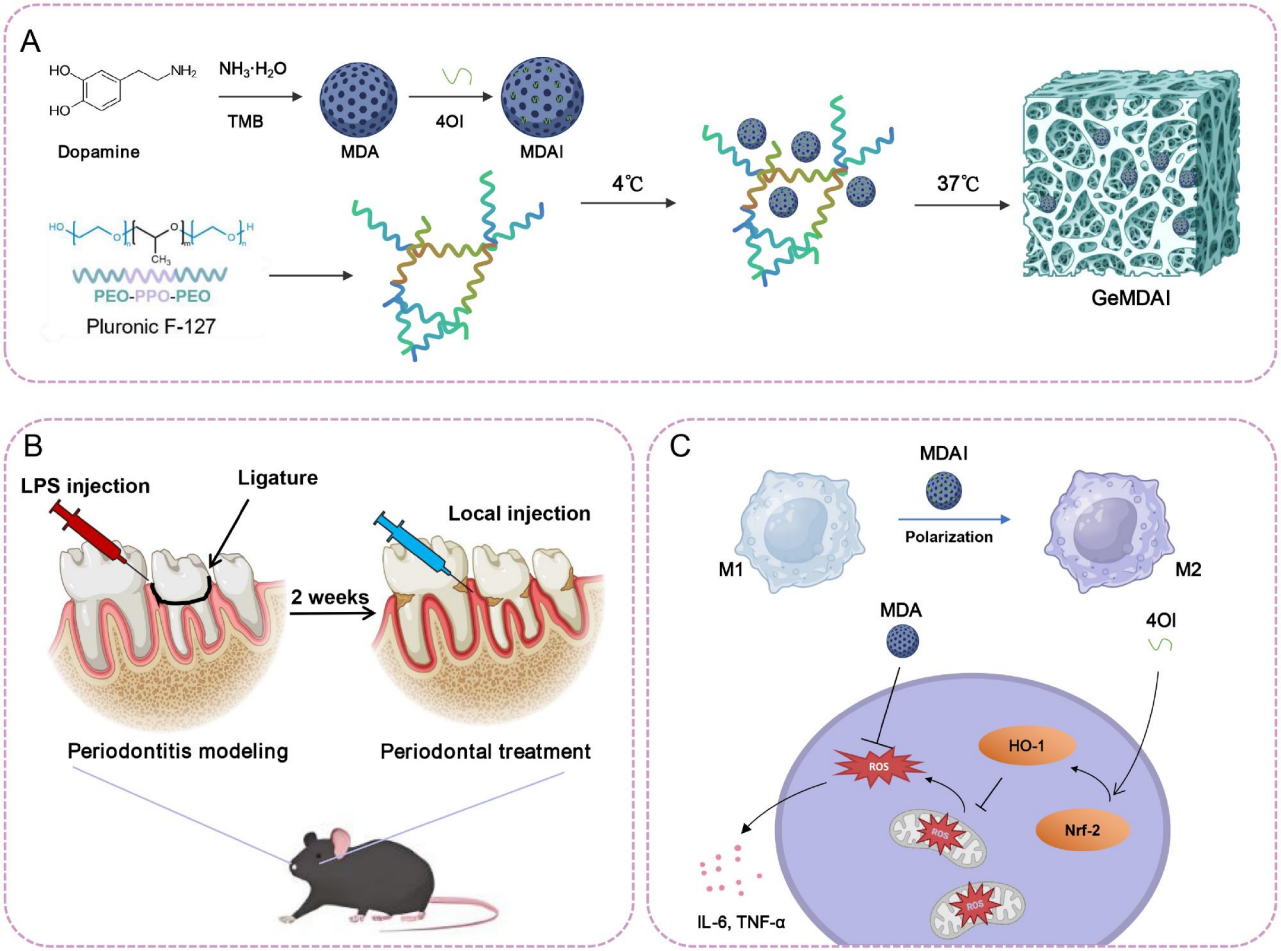
(**A**) Synthetic routes to MDAI and GeMDAI. (**B**) Therapeutic protocol: after 2 weeks of experimental periodontitis, the MDAI-loaded thermosensitive hydrogel was injected directly into the periodontal pocket, where it instantly formed a gel at body temperature. (**C**) The mechanism of MDAI with tandem antioxidant protection: MDA rapidly neutralized the intracellular ROS, whereas the sustained-release 4OI triggered the Nrf-2/HO-1 pathway, amplifying cellular antioxidant capacity. This dual suppression of oxidative stress downregulated IL-6/TNF-α, shifted macrophages from the M1 to the reparative M2 phenotype, and stimulated alveolar-bone regeneration, thereby remodeling the periodontal microenvironment.

Based on these considerations, this study introduces a chemico-biological cascade therapeutic strategy that integrates responsive antioxidant nanoparticles with thermosensitive hydrogel delivery to achieve coordinated anti-inflammatory and osteogenic effects. The system functions through three synergistic processes: (1) a chemical catalytic cascade, in which intracellular ROS trigger degradation of the mesoporous polydopamine (MDA) scaffold, enabling simultaneous ROS scavenging and 4OI release; (2) a biological regulatory cascade, where the released 4OI activates the Nrf-2/HO-1 pathway in macrophages, reinforcing antioxidant defenses, driving M2 polarization and suppressing inflammatory cytokines; and (3) an immuno-osteogenic coupling cascade, whereby the reprogrammed immune environment enhances osteogenic differentiation and bone regeneration. The integration of these nanoparticles within a thermosensitive hydrogel enables *in situ* injection and prolonged retention in periodontal pockets, effectively overcoming anatomical barriers (Figure 1). Collectively, this hybrid platform reestablishes redox balance, mitigates inflammation and promotes tissue regeneration, representing a versatile and powerful therapeutic approach for periodontitis and other oxidative stress–driven chronic diseases.

## Materials and methods

### Materials

Dopamine hydrochloride, Pluronic F-127 (PF127) and 4OI were purchased from Merck and Co., Inc. (Kenilworth, NJ, USA); FITC-conjugated phalloidin, DAPI, tetramethylbenzidine (TMB) and methylene blue (MB) solution was purchased from Solarbio Science and Technology Co., Ltd (Beijing, China); CCK-8, Annexin V-FITC/PI, calcein-AM/PI and Reactive Oxygen Species Assay Kit were obtained from Beyotime Biotechnology (Shanghai, China). Macrophage colony-stimulating factor (M-CSF) was purchased from MedChemExpress LLC (Shanghai, China). Fetal bovine serum (FBS), alpha minimum essential medium, phosphate-buffered saline (PBS) and penicillin/streptomycin (PS) were obtained from GIBCO (Grand Island, NY, USA). Ficoll–Paque PLUS (density gradient separation medium) was purchased from GE Healthcare (Uppsala, Sweden). Pacific blue-anti-CD11b, FITC-anti-F4/80, APC-anti-CD86 and PE-anti-CD206 were provided by BioLegend, Inc. (San Diego, CA, USA). Primary antibodies against CD206, iNOS, IL-6, TNF-α, Nrf-2 and HO-1 were obtained from BOSTER Biological Technology Co., Ltd (Wuhan, China).

### Preparation of mesoporous dopamine nanoparticles (MDA) and 4OI-Loaded MDA (MDAI)

Dopamine hydrochloride (50 mg) and 100 mg of PF127 were dissolved in 50% ethanol and thoroughly mixed. Then, 300 μL of trimethylbenzene was added and the pH of the solution was adjusted to 7–8. After mixing, the solution was stirred overnight at room temperature and centrifuged at 12 000 rpm for 5 min, after which the lower layer of nanoparticles was collected. The nanoparticles were washed 2–3 times with anhydrous ethanol to obtain MDA.

To load 4OI, 20 mg MDA were dissolved in an aqueous solution, and 300 μL of the 4OI solution (10 mg/mL) was added under stirring at 4°C for 2 h, after which MDAI were collected via centrifugation.

### Biodegradation and drug release under hydrogen peroxide *in vitro*

Following resuspension in a 10 mM H_2_O_2_ solution, MDA and MDAI were collected at 1 h and 4 h post-treatment. The morphological changes were analyzed by transmission electron microscopy (TEM), and the supernatant of MDAI collected after 4 h was analyzed by dynamic light scattering (DLS) to monitor the change in particle size distribution.

To evaluate the drug-release kinetics in response to ROS within an inflammatory microenvironment, an oxidative environment mimicking *in vivo* inflammation was established. The drug-release profiles were subsequently determined under these simulated pathological conditions. MDAI was dispersed in an aqueous solution containing 10 mM H_2_O_2_ and placed in a dialysis bag, which was then immersed in 200 mL of the buffer solution. At predetermined time points, 5 mL of the solution was withdrawn to measure the released drug concentration, and fresh 5 mL of buffer solution was added to calculate the release efficiency.

### Hydrogen peroxide consumption test

For the H_2_O_2_ consumption test, 2 mg/mL MDAI solution was incubated with 2 mL of 1 mM H_2_O_2_ at room temperature. At predetermined time points (0.5, 2, 8, 12, 24 h), the collected solution was assayed using a hydrogen peroxide assay kit to determine the residual H_2_O_2_ concentration.

### Stability

The MDAI was re-suspended in PBS, and their size distribution and polydispersity index (PDI) were measured every 24 h using DLS for a total of 120 h.

### Hydroxyl radical-scavenging assay

A solution of MDAI or MDA (2.5 mL of 4 mg/mL) was mixed with 1.25 mL of 6 mM H_2_O_2_ and 1.25 mL of 60 μM FeSO_4_ at room temperature for 10 min. Then, TMB or MB was added, and the mixture was stirred for 15 min. The absorbance was measured by a UV–Vis spectrophotometer.

### DPPH· and ABTS·+ scavenging assay

Different concentrations of MDAI were dissolved in 2 mL of aqueous solution, and 300 μL of 100 μM DPPH· solution was added to it. After stirring for 3 min, the solution was centrifuged to remove MDAI and the absorbance was measured by using a UV–Vis spectrophotometer. Meanwhile, the 2, 2ʹ-azino-bis (3-ethylbenzothiazoline 6-sulfonate) radicals (ABTS**·+**) assay kit was used to measure the oxidation resistance of the MDAI.

### Preparation of PF127 hydrogel (gel) and hydrogel-encapsulated MDAI (GeMDAI)

PF127 (1.8 g) was dissolved in deionized water and the volume was made up to 10 mL, followed by stirring overnight at 4°C. To prepare GeMDAI, PF127 was dissolved in MDAI solution, followed by stirring for an additional 12 h to obtain the MDAI-loaded hydrogel solution. Upon a rise in temperature, the hydrogel solution undergoes gelation into Gel and GeMDAI hydrogels.

### Cell sources and culturing

Primary mouse bone marrow-derived stem cells (BMSCs) and bone marrow-derived macrophages (BMDMs) were isolated from the femurs and tibias of 6-week-old C57BL/6 mice. The bone marrow cells were flushed out with α-MEM medium and processed separately for the two cell types. For BMSC isolation, the harvested cells were cultured in complete α-MEM medium supplemented with 10% FBS. After 48 h of culture, the non-adherent cells were removed by replacing the medium. The adherent BMSCs were subsequently expanded and used for experiments between passages 3 and 5. For BMDM isolation, the bone marrow cells were subjected to Ficoll density gradient centrifugation. Briefly, the cell suspension was layered over Ficoll solution and centrifuged at 400 × *g* for 30 min. The buffy coat containing mononuclear cells was carefully collected, washed twice with PBS, and then, resuspended in complete medium supplemented with M-CSF for differentiation.

Primary mouse gingival fibroblasts (GFs) were isolated from the gingival tissues of healthy mice. The tissues were minced into small fragments and digested with a solution of 0.2% collagenase-type I and 0.25% trypsin at 37°C for 30 min. The released cells were then collected via centrifugation and cultured in complete α-MEM medium supplemented with 10% FBS.

Murine macrophage cell line RAW264.7 was obtained from the Cell Bank of the Chinese Academy of Sciences (Shanghai, China) and maintained in DMEM containing 10% FBS.

All cells were cultured at 37°C in a humidified atmosphere with 5% CO_2_, and the medium was changed every 2–3 days.

### Cytotoxicity

A CCK-8 assay was utilized to assess the cytocompatibility of the 4OI, MDA, MDAI on RAW 264.7 cells, GFs and BMSCs. Cells were seeded in 96-well plates and, after attachment, treated with the test substances or NAC (a traditional antioxidant used as a control) for 1, 2 or 3 days. Cell viability was then quantified following the kit protocol. Similarly, the cytotoxicity of Gel and GeMDAI was also evaluated using CCK8.

To further measure the apoptosis, RAW264.7 cells were seeded into a 12-well plate and incubated overnight. After removing the culture medium, the MDAI solution was added and the cells were incubated for an additional 24 h before collection. The cells were stained with Annexin V-FITC and PI to analyze apoptosis and necrosis by flow cytometry. Concurrently, the aforementioned cells were stained with calcein-AM/PI and imaged by using a confocal laser scanning microscope (CLSM).

### Hemolysis assay

Peripheral blood from C57BL/6J was collected and diluted with PBS. Different concentrations of the MDAI solution were added, and the mixture was incubated at 37°C for 15–30 min. The extent of hemolysis was analyzed by centrifugation.

### Cellular uptake assay

The RAW264.7 cells were seeded into confocal microscopy dishes and incubated until reaching 90% confluence. Cy5-labeled MDAI was added, and the cells were incubated for 12 h. After removing the medium and washing with PBS 2–3 times, the cells were fixed with 4% paraformaldehyde and stained with FITC-conjugated phalloidin and DAPI. The cellular uptake was observed using CLSM. In addition, the cells were washed, collected and analyzed by flow cytometry.

### Detection of intracellular ROS and mitochondrial membrane potential

RAW264.7 cells were seeded into confocal microscopy dishes and incubated overnight. Then, 10 ng/mL LPS was added to incubate for 24 h, followed by different treatments [31]. After 24 h, the medium was removed, and the cells were washed with PBS 2–3 times. DCFH-DA probe (final concentration 1 μM) was added, and the cells were incubated at 37°C for 15–30 min. After washing with PBS, the cells were collected, and oxidative stress was assessed using both CLSM and flow cytometry.

To further investigate the extent of mitochondrial impairment, the cells subjected to the aforementioned treatments were stained with a JC-1 probe and analyzed via CLSM for alterations in red/green fluorescence intensity ratios.

### Real-time quantitative PCR analysis

Procedures were performed as previously described [32]. In brief, total RNA from each sample was extracted using TRIzol reagent, followed by reverse transcription with a cDNA synthesis kit. Quantitative real-time PCR (RT-qPCR) was performed using SYBR Green quantitative PCR master mix on a real-time PCR system (Thermo Fisher Scientific). The relative mRNA expression levels were calculated using the 2−ΔΔCt method. Primer sequence is listed below. NFE2L2 Forward: AGC CCAGCA CAT CCA GTC A, Reverse: CAG TCATCAAAGTACAAAGCATCTGA; HMOX1 Forward: CCAGGCAGAGAATGCTGAGTTC, Reverse: AAGACTGGGCTCTCCTTGTTGC; GAPDH Forward: GCCTCGTCTCATAGACAAGATGGT, Reverse: GAAGGCAGCCCTGGTAACC.

### Macrophage polarization assay

BMDMs were seeded into 24-well plates to incubate for a certain period of time. Then, BMDMs were subjected to different treatments for 24 h. The supernatant was collected, and the levels of TNF-α and IL-6 were detected by ELISA. The adherent cells were stained with live/dead dyes and then stained with antibodies of pacific blue-anti-CD11b, FITC-anti-F4/80, APC-anti-CD86 and PE-anti-CD206. Further immunofluorescence staining was performed for CD206, and polarization was observed using a CLSM. Meanwhile, the expression of CD86 and CD206 was analyzed by flow cytometry.

### RNA sequencing analysis

RNA sequencing (RNA-seq) was performed to identify differentially expressed genes (DEGs) in bone marrow–derived macrophages (BMDMs) under different conditions. Total RNA was extracted using TRIzol reagent (Invitrogen, California, USA) as per the manufacturer’s instructions. RNA integrity was assessed by agarose gel electrophoresis, and RNA quality was quantified using an Agilent 5300 analyzer based on the RNA Quality Number (RQN). Purified libraries were size-selected, PCR-amplified and sequenced on the Illumina NovaSeq 6000 platform (Shanghai Majorbio Bio-Pharm Technology Co., Ltd, China). Data processing and analysis were conducted using the Majorbio Cloud Platform. DEGs were identified with the DESeq2 package after applying a significance threshold of *P *< 0.05. Functional annotation and pathway enrichment analyses of DEGs were conducted via the GO and KEGG databases to clarify their involved biological processes and pathways.

### Osteogenic differentiation analysis

The osteogenic effect of the conditioned medium from MDAI-treated BMDMs on BMSCs was evaluated using alkaline phosphatase (ALP) and Alizarin Red S (ARS) staining. Briefly, LPS- stimulated BMDMs were treated with MDAI for 3 days. The resulting conditioned medium was then collected and applied to BMSCs undergoing osteogenic induction. The control group consisted of BMSCs treated only with the standard osteogenic induction medium (containing 10 mM β-glycerophosphate, 200 μM ascorbic acid and 100 nM dexamethasone). ALP staining was performed on day 14, and ARS staining was performed on day 28 of culture.

### *In vivo* retention

MDAI labeled with Cy5 was subsequently encapsulated within the hydrogel and then injected into the periodontal region of mice with periodontitis. Fluorescence images of the mice were acquired at predetermined time points (0.5, 2, 12, 24, 48, 96 and 144 h). Quantitative analysis of fluorescence intensity in the mouse periodontal region was performed to evaluate the sustained and controlled release efficacy of the hydrogel. In this experiment, free MDAI (not encapsulated in hydrogel) served as the control.

### Therapeutic effects of GeMDAI on mice with periodontitis

The study protocol involving animals was reviewed and approved by the Animal Ethics Committee of Shanghai Tongji Stomatological Hospital and Dental School (Ethical approval number: 2025-DWYY-042). All experiments were conducted in strict adherence to the ARRIVE guidelines and the National Institutes of Health (NIH) Guide for the Care and Use of Laboratory Animals.

With reference to previous literature [33], a periodontitis model was established by ligating the upper second molars with a 4–0 silk suture and injecting with LPS (200 μg/mL, 20 μL). The stability of the ligature was examined periodically. The clinical symptoms of periodontitis, such as gingival swelling, bleeding and periodontal pocket formation, indicated successful induction. After 2 weeks of the ligature, the ligature was removed, and the animals were randomly assigned to the following five groups: Control, Periodontitis, 4OI, MDAI and GeMDAI, with 5 mice each. The treatment regimen lasted for a total of 4 weeks (weeks 3–6). The Control group did not receive any treatment, whereas the Periodontitis group received 20 μL of saline, and each formulation group was administered 20 μL in the periodontal pocket every 7 days. All animals were euthanized at the end of the experiment.

To further evaluate the therapeutic effects of GeMDAI on mice with periodontitis, micro-CT scanning was performed: the maxilla was dissected, fixed with 4% paraformaldehyde, and scanned by using a micro-computed tomography (micro-CT) system (Scanco Medical, Zurich, Switzerland) at a resolution of 10 μm. Three-dimensional images were then reconstructed using Start Xming software, and the second molar area was selected as the region of interest (ROI) for analysis. The bone volume/total volume (BV/TV) were calculated. The distance between the cemento–enamel junction (CEJ) and the alveolar bone crest (ABC) was determined.

Next, the tissue samples were decalcified with 10% EDTA solution for 1 month, dehydrated with graded ethanol, processed with xylene and embedded in paraffin. The sections from the extraction site were stained with hematoxylin–eosin (HE) and Masson’s trichrome stain (MT). Tartrate-resistant acid phosphatase (TRAP) staining was performed by using an acid phosphatase kit (Sigma-Aldrich) to identify osteoclasts. Histological analysis was performed by using an optical microscope (Nikon Eclipse 80i).

In addition, the paraffin sections were deparaffinized, rehydrated and subjected to antigen retrieval in EDTA (pH 9.0) buffer. The sections were incubated overnight at 4°C with primary antibodies against CD206, iNOS, IL-6, TNF-α, Nrf-2 and HO-1 (1:100 dilution). After incubation with HRP-conjugated secondary antibody at room temperature for 1 h, DAB was used as a chromogen, followed by hematoxylin staining. ImageJ was used to analyze the positive area of CD206, iNOS, IL-6 and TNF-α.

### Statistical analysis

All quantitative data were performed as the mean ±SD. The statistical analysis was calculated by one-way analysis of variance (ANOVA), followed by Student’s *t*-test via GraphPad Prism 10.0 software. The label * was utilized to represent the statistical significance.

## Results

### Characterization of MDAI nanoparticles and hydrogel construction

**Figure 2.**
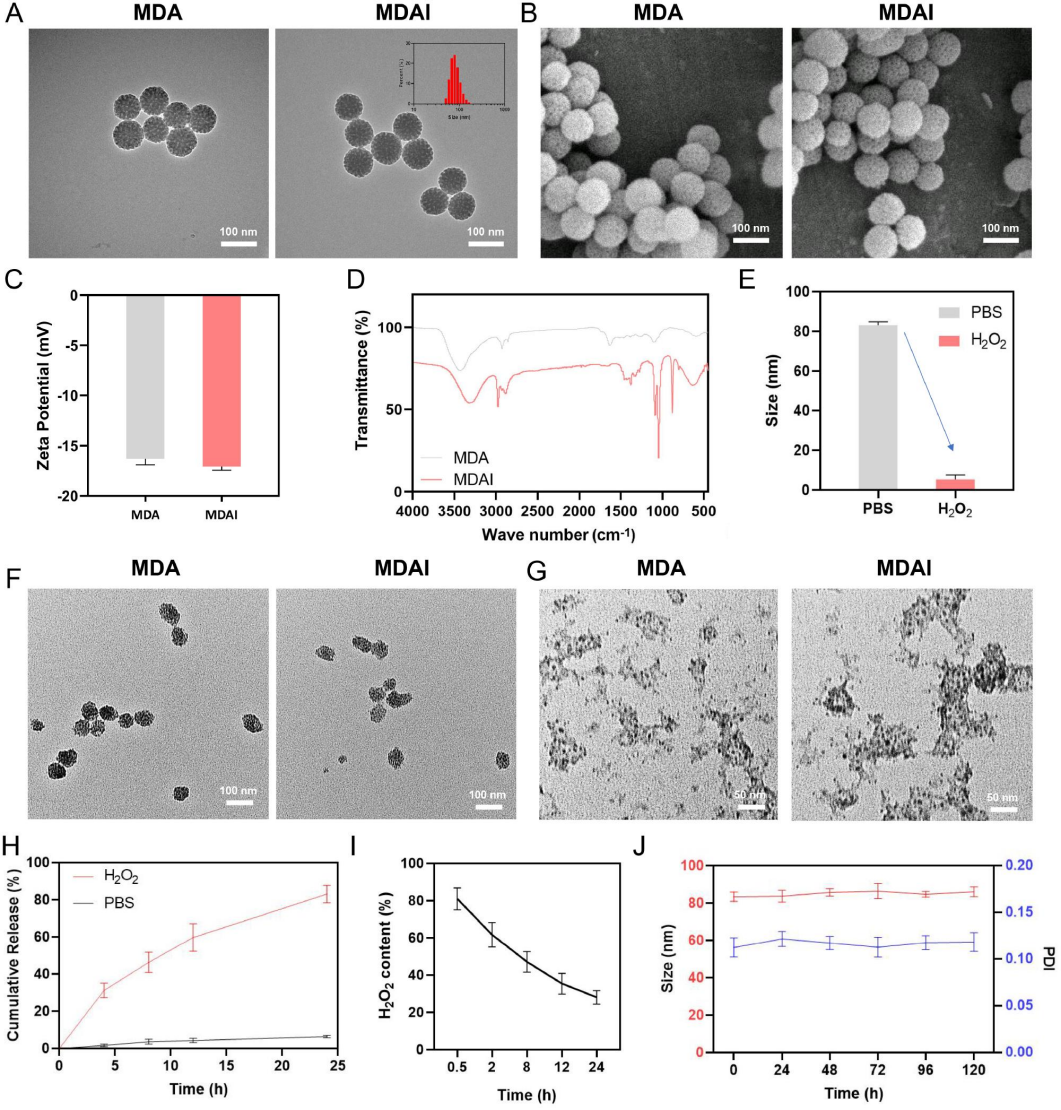
Characterization of MDA and MDAI nanoparticles. (**A**) Representative TEM images of MDA and MDAI (inset exhibited the size distribution of MDAI from DLS). (**B**) Representative SEM images of MDA and MDAI. (**C**) Zeta potential values of MDA and MDAI. (**D**) The structure of MDA and MDAI detected by FTIR. (**E**) The change in the size distribution of MDAI under different treatments. The morphology of MDA and MDAI under the stimulation of H_2_O_2_, observed at (**F**) 1 h and (**G**) 4 h post-incubation. (**H**) The release curve of 4OI under 10 mM H_2_O_2_. (**I**) Time-dependent H_2_O_2_ consumption. (**J**) The stability of MDAI by measuring the size distribution and PDI within 120 h.

As shown in Figure 2A, TEM imaging revealed that MDA and MDAI nanoparticles were uniformly dispersed and exhibited a consistent spherical morphology, with an average diameter of approximately 80 nm. Scanning electron microscopy (SEM) imaging (Figure 2B) further demonstrated that both MDA and MDAI nanoparticles possessed a loosely porous surface architecture, which is advantageous for efficient drug encapsulation. Analysis of the nanoparticle surface characteristics following 4OI incorporation showed minimal changes. After incorporation of 4OI, the zeta potential of MDAI remained nearly unchanged compared with MDA, maintaining a stable surface charge around −18 mV (Figure 2C). Collectively, these findings suggest that the incorporation of 4OI had negligible influence on the surface physicochemical properties of the nanoparticles. Fourier-transform infrared (FTIR) spectroscopy was performed to verify the chemical composition and bonding structure of the nanoparticles (Figure 2D). The broad band near 3300 cm^−1^ corresponds to O–H and N–H stretching [34]. Compared with MDA, the red-shifted and intensified band in MDAI indicates the formation of a dense hydrogen-bonding network between 4OI and the phenolic hydroxyl and amino groups of MDA. The absorption peaks at 2970 cm^−1^, 2930 cm^−1^ and 2880 cm^−1^ corresponded to asymmetric –CH_3_ stretching, asymmetric –CH_2_– stretching and symmetric –CH_3_ stretching [35]. The peak at 1050 cm^−1^ is predominantly due to the C–O stretching [32]. Compared with PBS, MDAI underwent relatively faster structural degradation when exposed to H_2_O_2_. After treatment with H_2_O_2_ for 1 h and 4 h, TEM imaging showed a slight collapse of the nanoparticle framework, accompanied by a relative decrease in particle size distribution after 1 h (Figure 2F). After 4 h, the nanostructure was partially disintegrated, and the fractured particles tended to aggregate. The persistence of MDAI structures after 4 h provides direct evidence of its incomplete degradation (Figure 2G). To avoid interference from particle aggregation, DLS measurements were performed on the supernatant of MDAI after 4 h of H_2_O_2_ treatment (Figure 2E). The *in vitro-*drug release profile is shown in Figure 2H. Under physiological conditions (PBS, 24 h), only approximately 10% of the encapsulated 4OI was released, demonstrating minimal passive diffusion. In contrast, the presence of H_2_O_2_ triggered a rapid and substantial release of 4OI, indicating that MDAI effectively responds to oxidative stress, a characteristic feature of the periodontitis microenvironment. The results of the H_2_O_2_ consumption assay demonstrated that the H_2_O_2_ concentration decreased over time during the degradation process of MDAI (Figure 2I). Additionally, the colloidal stability of MDAI NPs was assessed by resuspending them in PBS and monitoring size distribution and PDI over 120 h (Figure 2J). Both the parameters remained stable, demonstrating excellent dispersion stability and long-term physicochemical robustness in aqueous environments.

**Figure 3.**
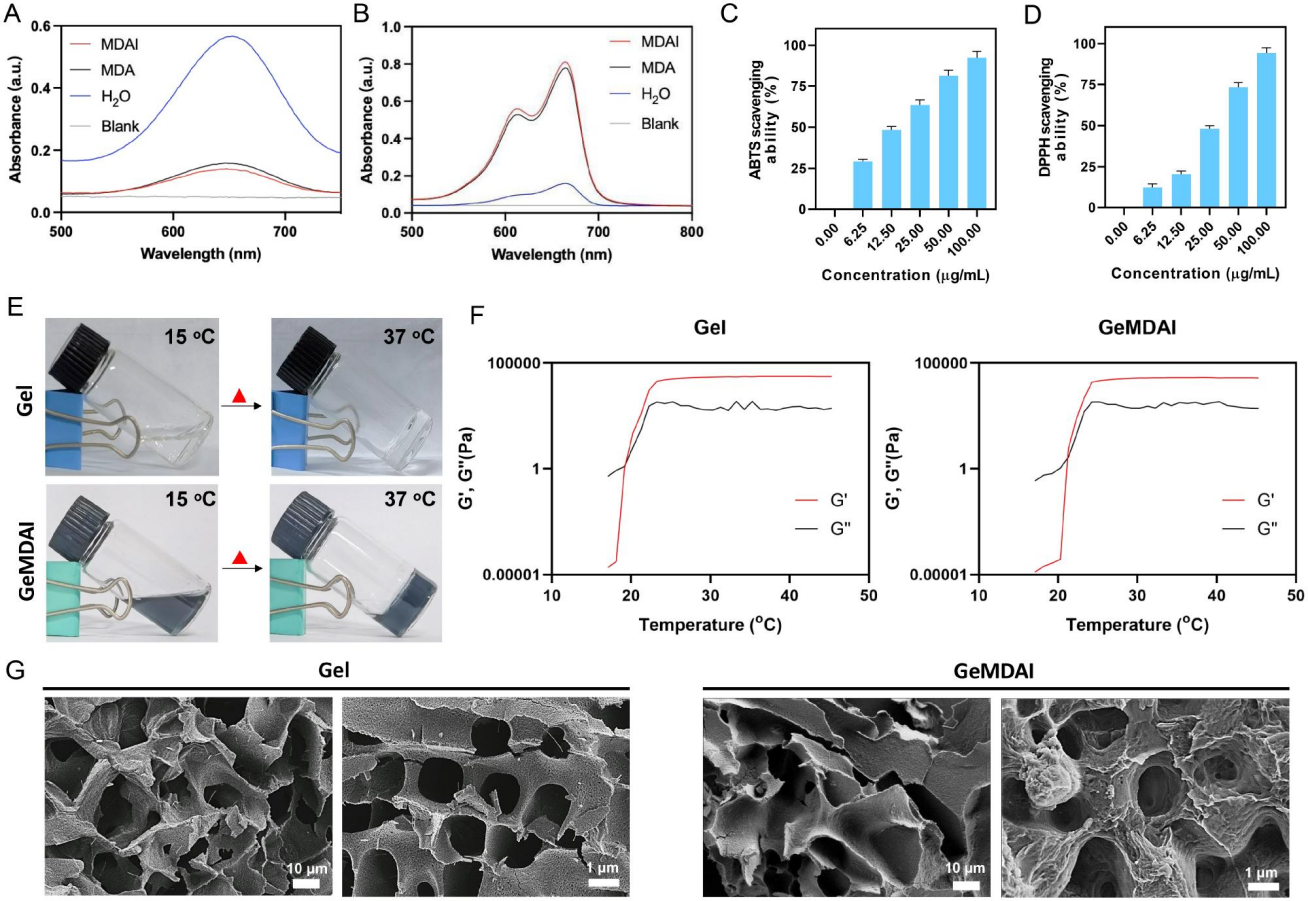
UV–Vis absorption spectra of (**A**) TMB and (**B**) MB with MDAI and MDA in the presence of Fe^2+^/H_2_O_2_. (**C**) ABTS·+ and (**D**) DPPH· radical scavenging rates of MDAI with different concentrations. (**E**) Sol–gel transition of GeL and GeMDAI. (**F**) Temperature-dependent rheology (G′, G″) of gel and GeMDAI. (**G**) The morphology of gel and GeMDAI observed by SEM.

As shown in Figure 3A, MDAI effectively catalyzed the oxidation of TMB while promoting MB decolorization (Figure 3B), confirming its hydroxyl radical-scavenging capability. This antioxidant property was further quantified using ABTS·+ and DPPH· assays, in which MDAI exhibited dose-dependent scavenging behavior (Figure 3C and D), demonstrating consistent and potent radical elimination activity across different ROS.

The thermosensitive behavior of Gel and GeMDAI hydrogels was then investigated. As temperature increased, the system underwent a reversible sol–gel transition, confirming its thermal responsiveness (Figure 3E). Incorporation of MDAI into the hydrogel matrix did not disrupt this phase transition behavior, as evidenced in Figure 3F, indicating that the nanoparticles did not compromise the intrinsic gelation characteristics or structural integrity of the hydrogel. To further evaluate the viscoelastic properties relevant to periodontal application, rheological tests were performed on both Gel and GeMDAI. Temperature sweep results (Figure 3F) revealed a clear sol–gel transition, with the storage modulus (G′) surpassing the loss modulus (G″) around 20°C, defining the critical gelation temperature. Below this point, the material remained in a low-viscosity sol state (G″ > G′), suitable for injection; above it, a stable gel network formed (G′ > G″), enabling adhesion under oral physiological conditions. SEM imaging was used to examine the microstructure of the hydrogels (Figure 3G). Both Gel and GeMDAI displayed a continuous porous architecture, with no significant morphological difference observed after nanoparticle incorporation. This further supports that MDAI integration preserves the structural consistency of the hydrogel network.

### Evaluation of the biosafety and cell uptake of MDAI

**Figure 4.**
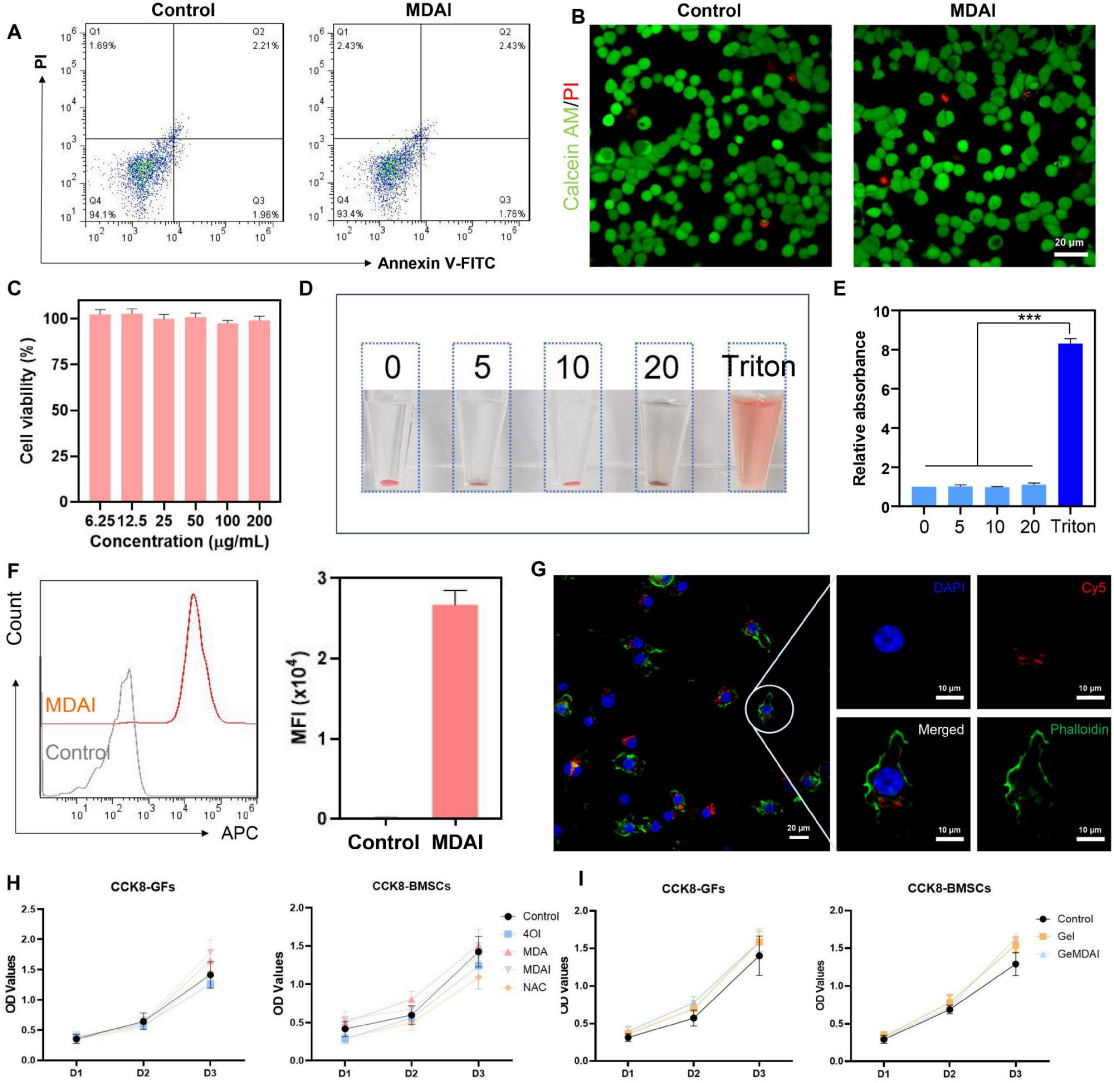
(**A**) Detection of apoptosis and necrosis in RAW264.7 cells treated with MDAI. (**B**) Macrophages treated with MDAI stained with calcein-AM/PI, observed by CLSM. (**C**) Cell viability after incubation with different concentrations of MDAI of RAW264.7 cells. Hemolysis assay of MDAI by incubating blood with different concentrations of MDAI, (**D**) the images captured by camera and (**E**) the relative absorption at 561 nm. (**F**) The uptake of MDAI labeled with Cy5 fluorescence after interaction with RAW264.7 cells via flow cytometry and the statistical graphs of MFI. (**G**) Image of MDAI endocytosed by RAW264.7 cells captured by CLSM. (**H**) Cell viability detected by CCK8 assay of GFs and BMSCs. (**I**) Cell viability detected by CCK8 assay of GeL and GeMDAI. (***P < 0.001).

Before *in vivo* testing, the cytocompatibility and cellular uptake of MDAI were evaluated *in vitro* using RAW264.7 macrophages. Flow cytometry was employed to quantify apoptotic and necrotic cells. As shown in Figure 4A, treatment with MDAI did not induce significant apoptosis or necrosis, confirming its favorable cytocompatibility. Live/dead staining results (Figure 4B) were consistent, further verifying that MDAI treatment did not cause noticeable cytotoxic effects. Moreover, dose-dependent viability assays (Figure 4C) demonstrated that RAW264.7 cells maintained normal morphology and proliferation across a range of MDAI concentrations, underscoring its biosafety. Hemocompatibility was also assessed through hemolysis testing. As shown in Figure 4D, MDAI-induced negligible hemolysis across the tested concentration range. Quantitative analysis of the supernatant absorbance (Figure 4E) confirmed these findings, further supporting the excellent biocompatibility of the nanoparticles. To examine cellular internalization, MDAI NPs were fluorescently labeled with Cy5 and incubated with RAW264.7 cells. Flow cytometry results revealed strong intracellular fluorescence signals, indicating efficient cellular uptake (Figure 4F). CLSM images (Figure 4G) corroborated these findings, clearly visualizing the intracellular distribution of Cy5-labeled MDAI. These results collectively demonstrate that MDAI is readily internalized by macrophages, providing a solid foundation for subsequent biological activity and therapeutic function.

Given the importance of GFs and BMSCs in the periodontal environment, we performed CCK-8 assays to fully assess the toxicological profile of MDAI nanoparticles and GeMDAI. The results (Figure 4H and I) showed that cell viability across all treatment groups matched or exceeded that of the Control. Notably, the MDAI group significantly promoted proliferation in both BMSCs and GFs by day 3, indicating bioactivity beyond basic biocompatibility. Furthermore, MDAI demonstrated a broad protective effect by effectively mitigating the mild cytotoxicity induced in BMSCs by either 4OI or the established antioxidant NAC.

### *In vitro* mechanism exploration of antioxidant activity

**Figure 5.**
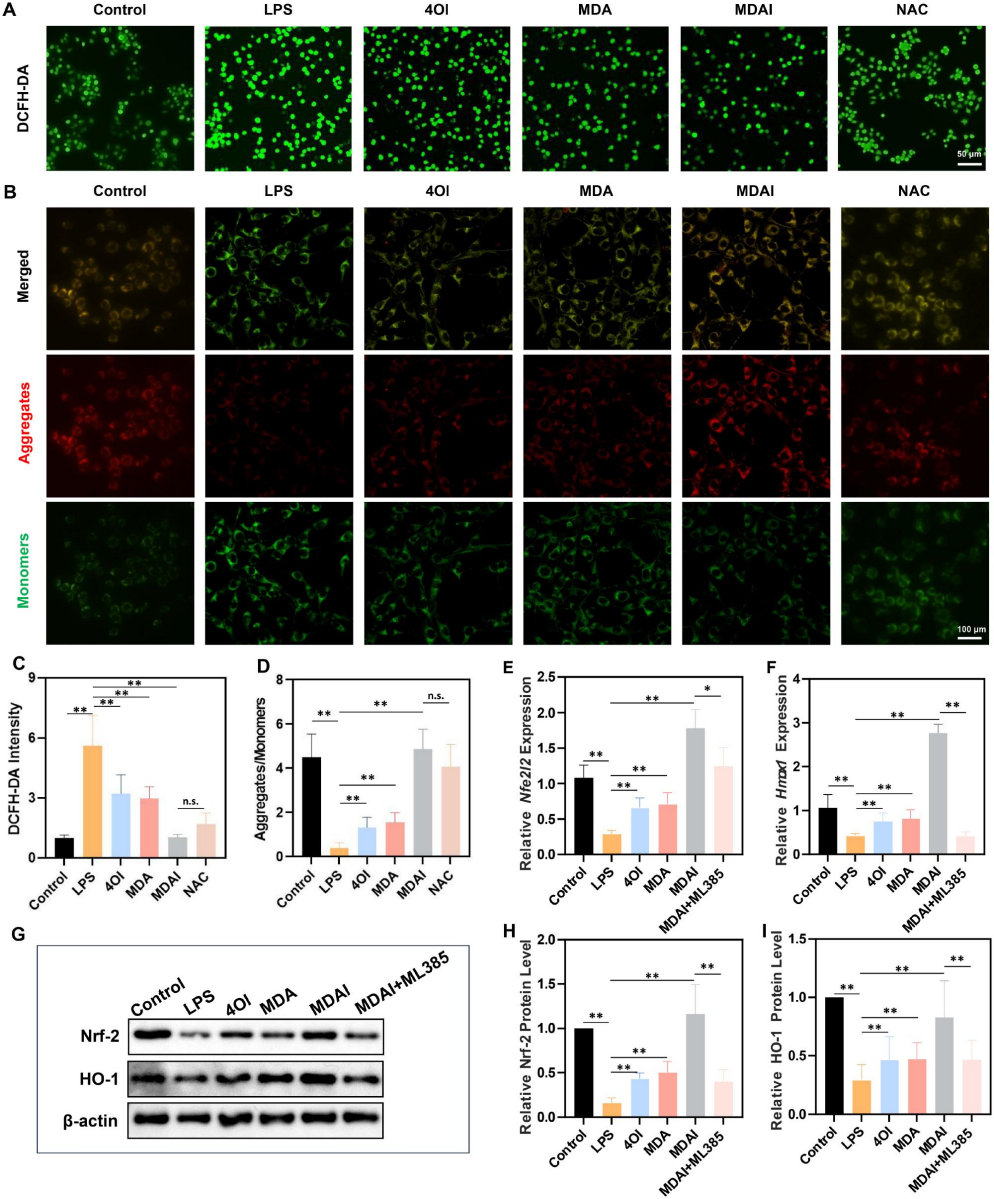
Evaluation of the ROS-scavenging ability of MDAI *in vitro*. (**A**) RAW264.7 cells were treated with LPS, and then, subjected to different treatments, followed by observation with a ROS probe DCFH-DA. (**B**) Measurement of mitochondrial membrane potential in macrophages under different treatments, as visualized by CLSM. (**C**) DCFH-DA fluorescence intensity. (**D**) JC-1 aggregates/monomers ratio across treatments. Relative mRNA expression of (**E)** *Nfe2l2* and (**F**) *Hmox.* (**G**) Western blot analysis of Nrf-2 and HO-1. Relative protein levels of (**H**) Nrf-2 and (**I**) HO-1. (*P < 0.05, **P < 0.01, ns: not significant).

Following confirmation of efficient internalization of MDAI by RAW264.7 macrophages, the nanoparticles’ ability to modulate intracellular ROS levels was examined. As shown in Figure 5A, treatment with MDAI led to a noticeable decrease in ROS-associated fluorescence, indicating reduced oxidative stress. Quantitative analysis corroborated these observations, demonstrating a consistent reduction in the ROS levels compared to controls (Figure 5C). The mitochondrial membrane potential was assessed using JC-1 staining. MDAI treatment resulted in a marked increase in red fluorescence, and decrease in green fluorescence, signifying the restoration of mitochondrial membrane potential and improvement of cellular energy status (Figure 5B and D). Additionally, compared to the conventional antioxidant NAC, MDAI also exhibited a more potent capacity to suppress ROS overproduction and prevent the collapse of mitochondrial membrane potential. To elucidate the underlying molecular mechanism, we analyzed the expression of key genes and proteins in the Nrf-2 pathway. MDAI treatment significantly upregulated the mRNA levels of *Nfe2l2* and *Hmox1*, which encode Nrf-2 and HO-1 proteins, respectively (Figure 5E and F). Consistently, it also elevated the protein levels of Nrf-2 and HO-1, effectively rescuing their LPS-induced suppression (Figure 5G–I). This suggests that MDAI mitigates oxidative stress and subsequent inflammatory signaling by activating this pathway. The critical role of the Nrf-2/HO-1 axis was further confirmed using ML385, a specific Nrf-2 inhibitor. ML385 treatment abrogated the MDAI-induced upregulation of Nrf-2, leading to a consequent decrease in both Hmox1 mRNA and HO-1 protein levels. This result confirms that Nrf-2/HO-1 activation is essential for the ROS-clearing activity of MDAI.

**Figure 6.**
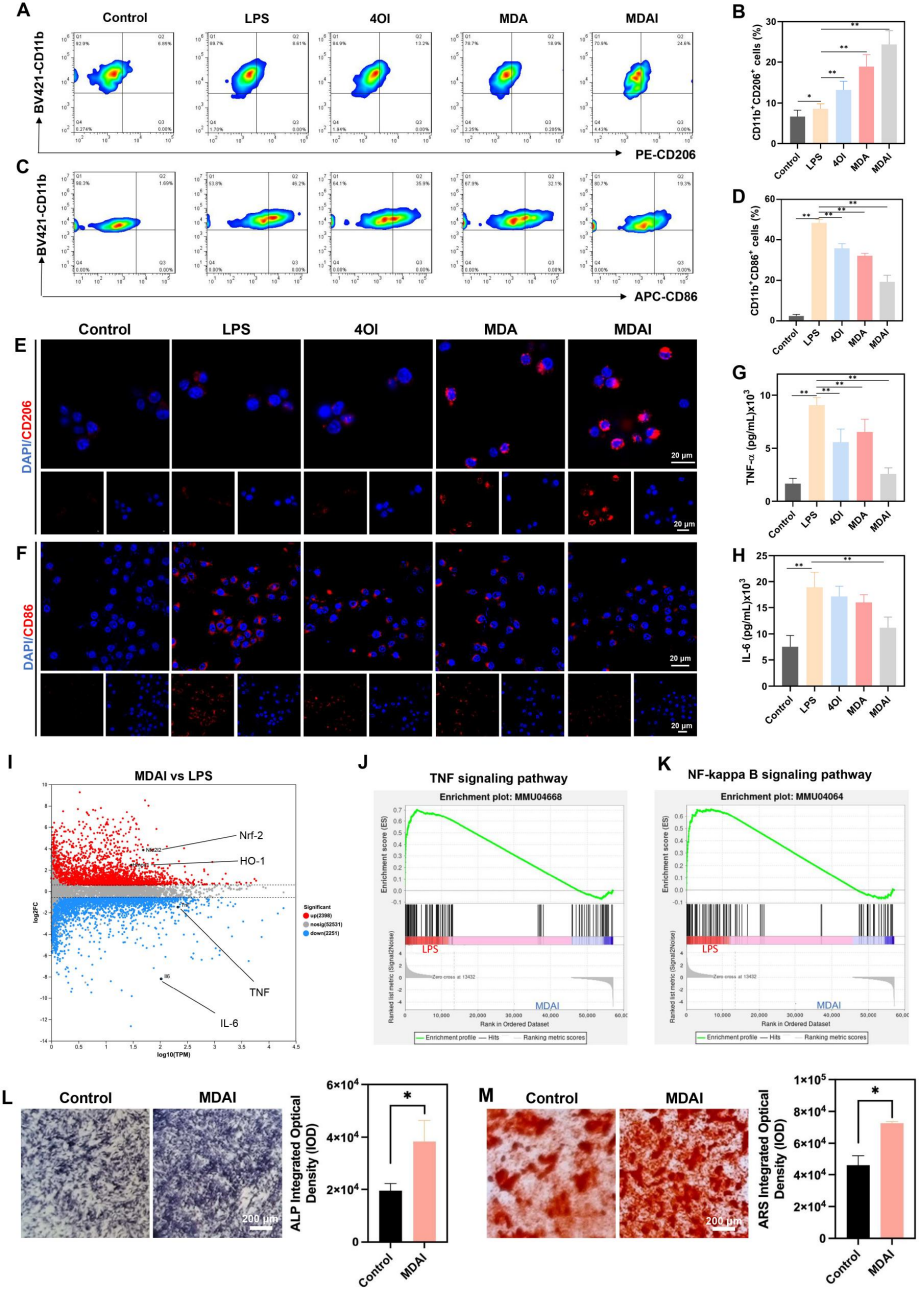
Evaluation of the macrophage-polarization effects of MDAI. BMDMs exhibited different levels of (**A**, **B**) CD206 and (**C**, **D**) CD86 expressions post-treatment, as measured by flow cytometry. The expressions of (**E**) CD206 and (**F**) CD86 observed by CLSM. Inflammatory cytokine (**G**) TNF-α and (**H**) IL-6 expressions in the culture medium. (**I**) Volcano heatmap exhibiting significantly upregulated and downregulated genes in LPS-induced BMDMs treated by MDAI. (**J**) TNF-signaling pathway or (**K**) NF-κb-signaling pathway. Osteogenic differentiation assessed by (**L**) ALP and (**M**) ARS staining. (*P < 0.05, **P < 0.01).

The impact of oxidative stress modulation on macrophage polarization was subsequently assessed. Flow cytometry revealed a pronounced shift toward the anti-inflammatory M2 phenotype following MDAI treatment (Figure 6A), with quantitative analysis showing a two-fold increase relative to the control group (Figure 6B). Confocal fluorescence imaging confirmed enhanced M2 marker expression, with the MDAI-treated group exhibiting the strongest signal (Figure 6E). Flow cytometry and immunofluorescence staining both indicated that the expression of CD86, a surface marker of the M1 phenotype, was significantly reduced in the MDAI-treated group (Figure 6C, D and F). Consistent with these phenotypic changes, cytokine measurements of the culture supernatant revealed significant reductions in pro-inflammatory mediators TNF-α and IL-6, demonstrating that MDAI both promotes M2 polarization and suppresses inflammatory cytokine secretion (Figure 6G and H). To further explore transcriptional regulation, LPS-stimulated BMDMs were treated with MDAI and subjected to transcriptomic sequencing. The resulting volcano plot (Figure 6I) highlighted downregulation of key pro-inflammatory genes, including TNF and IL-6. Gene set enrichment analysis and Kyoto Encyclopedia of Genes and Genomes pathway mapping revealed that MDAI treatment modulated TNF (Figure 6J) and NF-κB (Figure 6K) signaling pathways, confirming its capacity to dampen inflammatory signaling at the molecular level. Collectively, these findings demonstrate that MDAI exerts potent antioxidant effects that reduce oxidative stress, promote M2 macrophage polarization and suppress pro-inflammatory signaling.

### Effect of MDAI on osteogenic differentiation of BMSCs

Considering the critical role of osteogenic differentiation in bone regeneration, we investigated the effect of MDAI-treated conditioned medium on BMSCs cultured in conditioned medium from MDAI-treated BMDMs. ALP and ARS staining revealed that MDAI-treated conditioned medium markedly enhanced osteogenic potential. Specifically, ALP activity was significantly intensified, and calcium nodule formation, as indicated by ARS staining, showed robust increases compared with control groups (Figure 6L and M). These results indicate that MDAI indirectly promotes osteogenesis by remodeling the inflammatory microenvironment and supporting bone formation.

### *In vivo* retention

**Figure 7.**
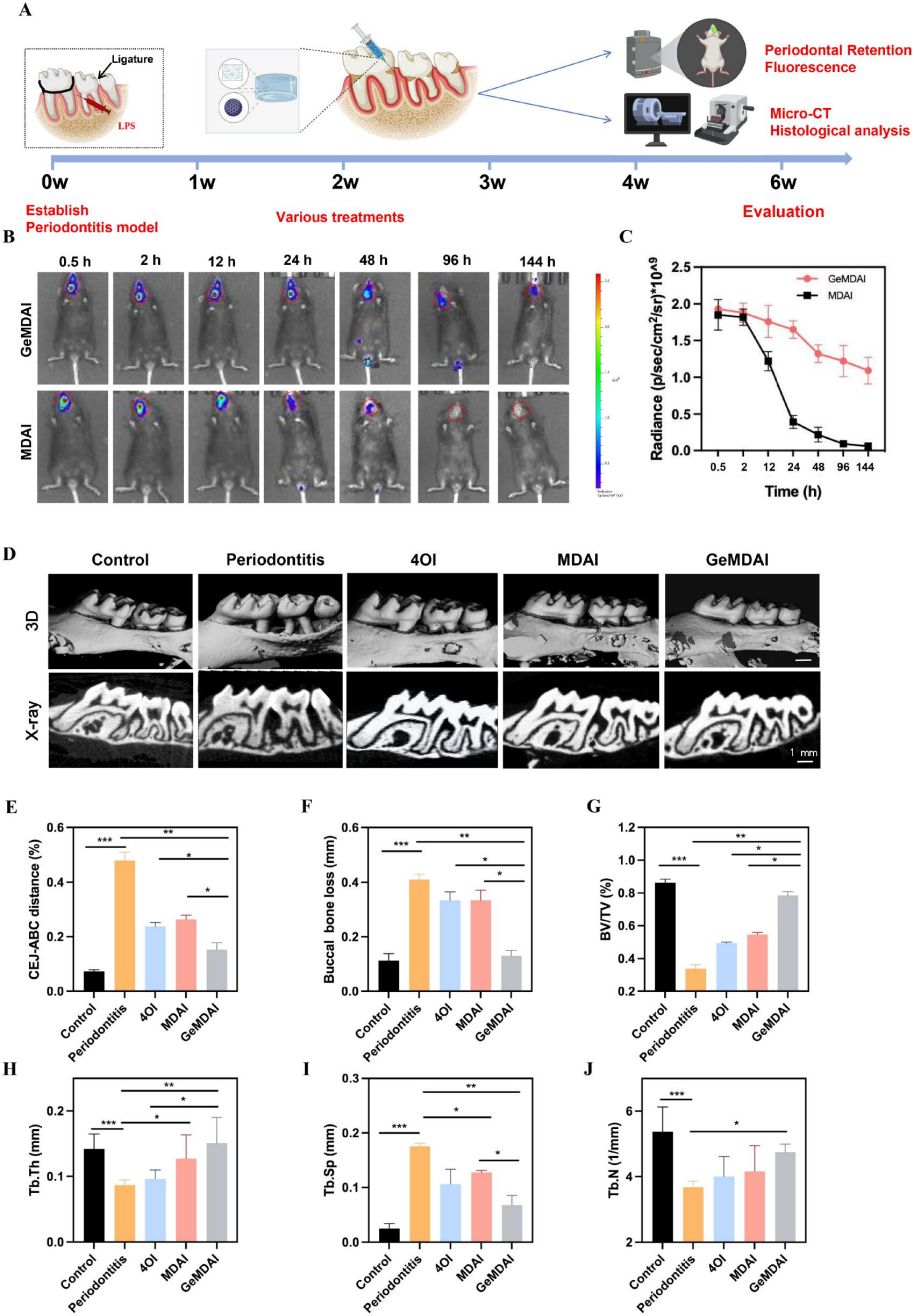
(**A**) Schematic illustration of the establishment of periodontitis in mice, and *in vivo* imaging and treatment of the lesion site. (**B**) Representative images of periodontitis mice injected with either fluorescently labeled MDAI and GeMDAI, captured at distinct time points, (**C**) Fluorescence quantification in the periodontal area (*n* = 3). Three-dimensional reconstruction and buccal–palatal sectional of maxillary molars and alveolar bone obtained via micro-CT scanning. (**D**) The two-dimensional and 3D reconstruction images for 4 weeks (*n* = 5, scale bar = 1 mm). Quantitative evaluation of (**E**) CEJ-ABC distance, (**F**) Buccal bone loss and (**G**) BV/TV. Quantitative analysis of bone morphological indicators (**H**) Tb.Th, (**I**) Tb.Sp and (**J**) Tb.N. (*P < 0.05, **P < 0.01, ***P < 0.001).

Prolonged retention at the site of inflammation is a prerequisite for achieving effective therapeutic concentrations *in vivo*. To evaluate this, an *in situ* retention assay was conducted using a murine periodontitis model (Figure 7A). Fluorescently labeled MDAI NPs, without or with thermosensitive hydrogel encapsulation, were locally injected into the periodontal lesions. Imaging at defined time points revealed that hydrogel-encapsulated MDAI exhibited sustained retention, with detectable fluorescence persisting for up to 7 days (Figure 7B). In contrast, unencapsulated MDAI, although initially localized, demonstrated rapid signal diminution over time, highlighting the role of the hydrogel in prolonging nanoparticle residence. Quantitative analysis of fluorescence intensity in the periodontal region confirmed these observations, showing a slower decay rate for hydrogel-embedded MDAI compared to free nanoparticles (Figure 7C). These results demonstrated that the hydrogel system effectively enhances local retention and supports sustained release, which is critical for maintaining therapeutic efficacy in periodontitis.

### *In vivo* therapeutic efficacy of GeMDAI

The procedure for constructing and administering the periodontitis mouse model is illustrated in Figure 7A. Representative micro-CT images of the maxilla, along with reconstructed 3D images of the upper second molar region after four weeks of treatment, are presented in Figure 7D. To quantify alveolar bone loss, the distance between CEJ and ABC was measured across all groups. Both PBS-treated and GeMDAI-treated groups exhibited varying degrees of bone resorption. Notably, quantitative analysis revealed that buccal bone defect height in the GeMDAI group (130 ± 20 μm) was significantly lower than that in the control group (410 ± 20 μm), and the CEJ–ABC distance in the GeMDAI group (153.3 ± 25.1 μm) was markedly shorter than that of the control (480 ± 30 μm; Figure 7E). Buccal bone defect measurements reflected disease severity, with the GeMDAI group (0.13 ± 0.02 mm) displaying significantly smaller defects than other treatment groups (*P *< 0.05) and comparable dimensions to the control group (0.11 ± 0.03 mm; *P *> 0.05; Figure 7F). BV/TV analysis further supported these findings. The GeMDAI group exhibited higher BV/TV values (78.35 ± 2.54%) than the control group (33.94 ± 2.39%) and approached levels observed in healthy mice (86.3 ± 2.1%; Figure 7G), indicating effective promotion of periodontal tissue regeneration. Additionally, trabecular analysis revealed that mean trabecular number in the GeMDAI group (4.75 ± 0.25 mm^−1^) surpassed other treatment groups while remaining slightly below the healthy control (5.30 ± 0.75 mm). Trabecular separation, reflecting marrow cavity expansion during bone resorption, was lowest in the GeMDAI group (0.07 ± 0.02 mm), nearly half that of the control (0.17 ± 0.01 mm). Correspondingly, trabecular thickness reached its maximum in the GeMDAI group (0.15 ± 0.04 mm), consistent with increased trabecular number, collectively indicating robust promotion of trabecular bone formation at alveolar defect sites (Figure 7H–J).

**Figure 8.**
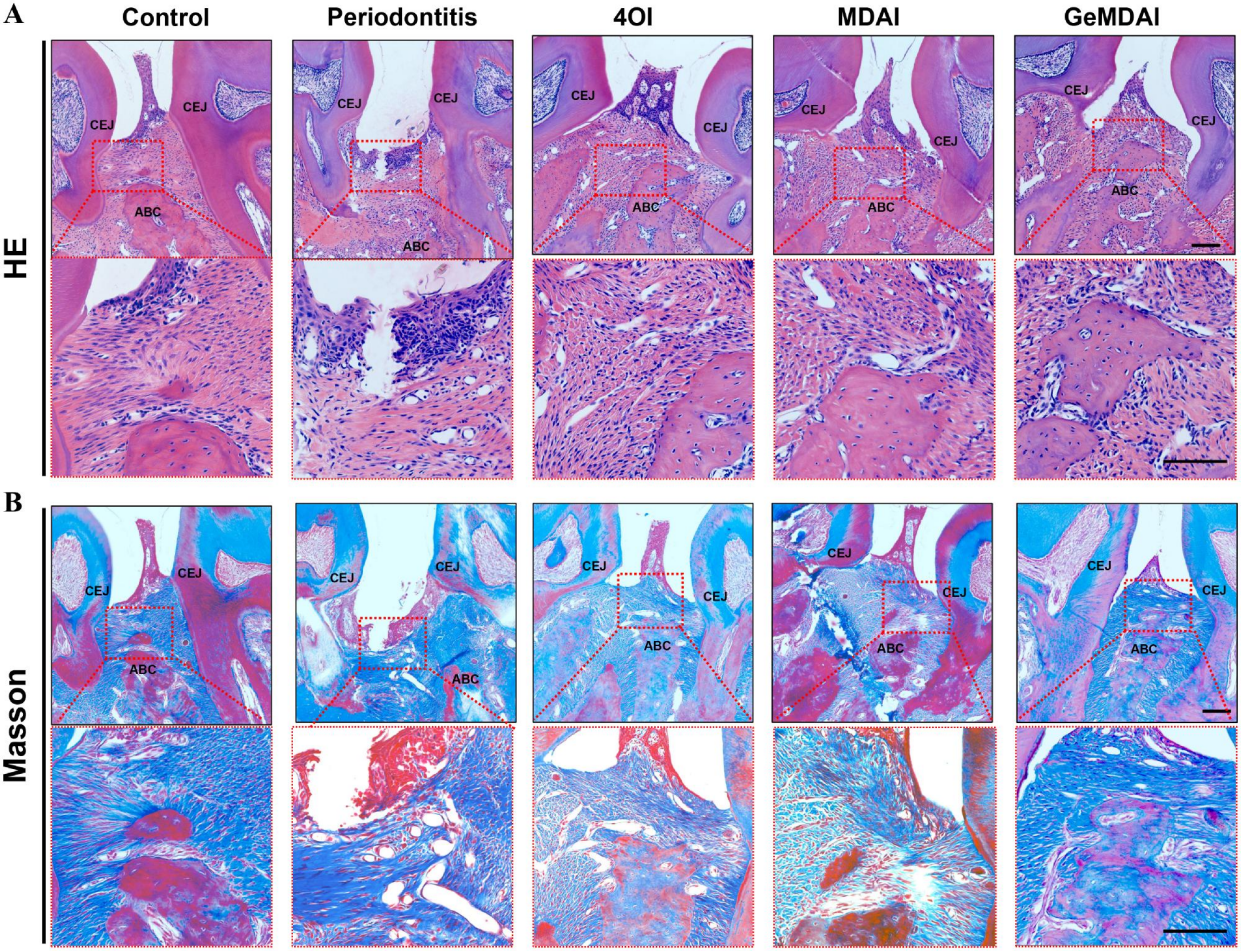
The bone tissues of mice with periodontitis were stained with (**A**) H&E and (**B**) Masson after different treatments. Scale bar = 100 μm.

**Figure 9.**
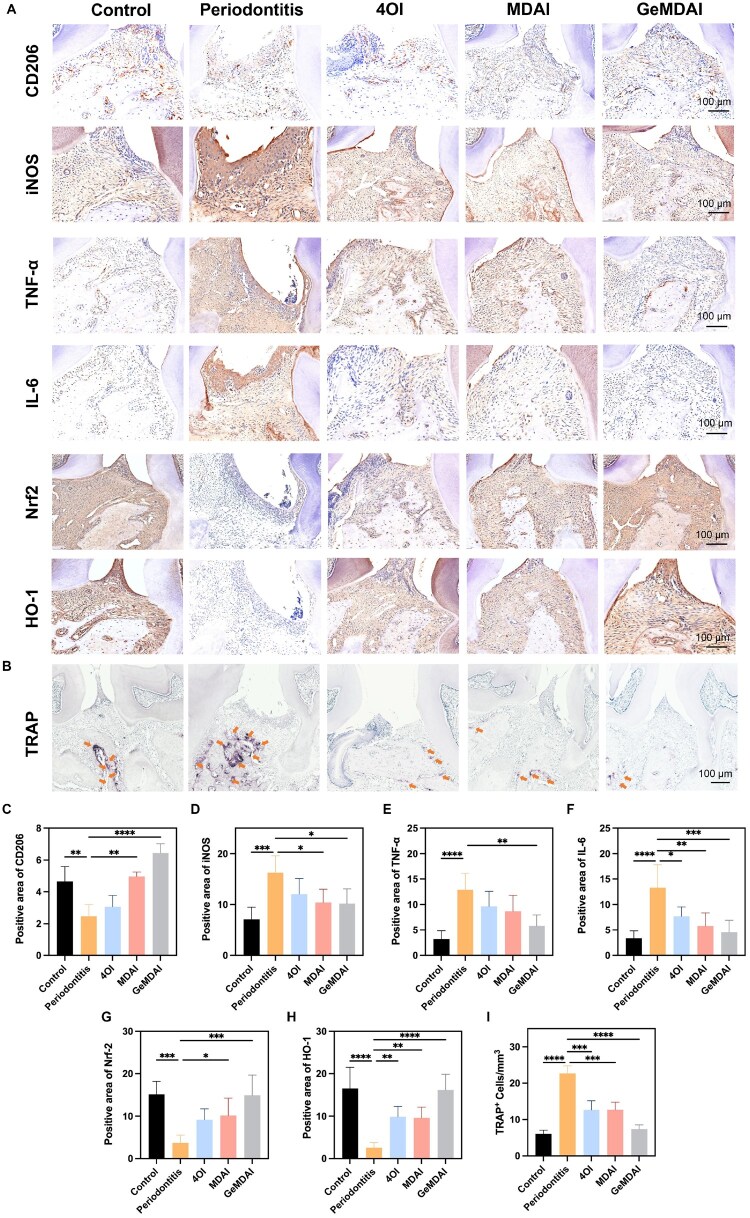
Periodontitis mice were immunohistochemically stained with (**A**) CD206, iNOS, TNF-α, IL-6, Nrf-2 and HO-1 after different treatments. (**B**) TRAP staining for osteoclasts identification (arrows indicating the osteoclasts). Quantitative evaluation of the expression of (**C**) CD206, (**D**) iNOS, (**E**) TNF-α, (**F**) IL-6, (**G**) Nrf-2, (**H**) HO-1 and (**I**) TRAP. Scale bar = 100 μm (*P < 0.05, **P < 0.01, ***P < 0.001, ****P < 0.0001).

Histological evaluation of decalcified maxillary specimens was conducted using H&E and Masson staining (Figure 8A and B). In the healthy group, alveolar bone resorption was negligible, whereas the control group exhibited substantial loss of bone height and absent interdental papillae, characteristic of periodontitis. In contrast, the GeMDAI group maintained significantly higher alveolar bone height, and epithelial morphology closely resembled normal tissue, with intact interdental papillae. MT staining revealed that regenerated bone in the GeMDAI group consisted predominantly of mature, organized bone and collagen deposition appeared dense and well-structured, reflecting enhanced soft tissue regeneration. TRAP staining (Figure 9B) demonstrated a significant reduction in TRAP-positive osteoclasts in the GeMDAI group relative to controls, indicating inhibition of osteoclast-mediated bone resorption (Figure 9I).

To further evaluate anti-inflammatory and osteogenic effects, immunohistochemical staining was performed for CD206, iNOS, TNF-α, IL-6, Nrf-2 and HO-1 (Figure 9A). GeMDAI treatment significantly upregulated CD206 expression while downregulating iNOS and IL-6 levels within periodontal tissues, indicating effective modulation of the inflammatory microenvironment and promotion of alveolar bone regeneration. These findings collectively suggest that GeMDAI exerts anti-inflammatory, osteoclast-inhibitory and osteogenesis-promoting effects, synergistically suppressing alveolar bone loss and representing a potent therapeutic strategy for periodontitis. Furthermore, TNF-α and IL-6, key mediators of inflammatory cascades, were minimally expressed in the healthy controls, exhibiting negligible brown staining [36]. In contrast, the periodontitis model displayed intense staining indicative of heightened inflammation. Treatment with GeMDAI substantially reduced cytokine expression, reflecting efficient suppression of inflammatory responses. Quantitative analysis confirmed these observations (Figure 9C–H). Collectively, these data demonstrate that GeMDAI mitigates inflammation, promotes M2 macrophage polarization, and shifts periodontal tissue from a pro-inflammatory state toward a reparative, anti-inflammatory phenotype. Nrf-2 is a key regulator of the endogenous antioxidant defense system [37]. Immunohistochemical analysis revealed significantly lower Nrf-2 expression in the periodontitis group compared with healthy controls, and HO-1 exhibited a similar staining pattern in periodontal tissues. Compared with the control group, the positive areas of Nrf-2 and HO-1 were significantly increased in the GeMDAI group, indicating an upregulation of protein expression. In summary, these results suggest that GeMDAI exerts superior anti-inflammatory effects *in vivo* by modulating the Nrf-2/HO-1 signaling pathway.

## Discussion

This study introduces a paradigm-shifting strategy for periodontitis therapy by implementing a spatiotemporally coordinated “ROS clearance–immune remodeling–regeneration” approach. Unlike conventional interventions that target individual pathological aspects, our multifunctional platform simultaneously addresses oxidative stress, immune dysregulation and tissue destruction through rationally engineered nanoparticles integrated with intelligent delivery matrices [6, 38–40].

MDA nanoparticles surpass traditional antioxidant nanoparticles or nanozymes (e.g. CeO_2-_, Cu- or diamond-based systems) that merely scavenge ROS [41–43]. By combining ROS scavenging with immunomodulatory drug delivery, MDA transitions from a passive antioxidant to a dynamic catalytic platform [44]. Unlike monofunctional ROS scavengers, MDA exhibits broad-spectrum peroxidase-like activity, efficiently catalyzing H_2_O_2_ decomposition and achieving over 90% scavenging efficiency for both ABTS·+ and DPPH· radicals. This dual functionality enables MDA to actively modulate the inflammatory microenvironment rather than simply neutralize ROS. The hierarchically porous architecture of MDA facilitates efficient encapsulation of the immune-metabolic modulator 4OI, overcoming the limitations of free 4OI, including rapid diffusion and short half-life. Structurally, the system demonstrates pronounced microenvironmental responsiveness. While stable under physiological conditions, the nanoparticles undergo oxidative cleavage in H_2_O_2_-rich pathological environments, triggering up to 83% 4OI release. This ROS-triggered release establishes a self-amplifying feedback loop: elevated ROS initiate drug liberation, and the released 4OI subsequently scavenges ROS. Beyond redox neutralization, 4OI reprograms macrophage metabolism via Nrf-2 activation, promoting a shift toward an anti-inflammatory phenotype. Collectively, this cascade converts MDAI from a passive carrier into an actively immune-modulating therapeutic platform.

To address rapid drug clearance from dynamic periodontal pockets, we incorporated a thermoresponsive PF127 hydrogel. The hydrogel undergoes a reversible sol–gel transition at physiological temperature (37°C) and provides three key advantages. First, it prolongs local retention of MDAI for up to 144 h, counteracting washout by gingival crevicular fluid, a major limitation of local periodontal drug delivery. Second, it enables controlled release kinetics, suppressing the initial burst (15% within 24 h) while permitting ROS-triggered 4OI release to sustain macrophage reprogramming. Third, the injectable hydrogel conforms to the irregular geometries of periodontal pockets, ensuring clinical practicality. Future work will need to evaluate the long-term stability of this system under lysozyme-rich conditions present in periodontal tissues.

Mechanistically, this platform restores periodontal homeostasis by targeting ROS-driven inflammation. Previous studies have established that Nrf-2 serves as a master regulator of cellular antioxidant defense and plays a pivotal role in maintaining mitochondrial integrity, including the stabilization of mitochondrial membrane potential [45, 46]. Upon activation, Nrf-2 translocates into the nucleus and upregulates HO-1-an enzyme that degrades pro-oxidant heme and generates biliverdin and bilirubin, both of which exhibit ROS-scavenging capacity [47]. Consistent with this mechanism, our data show that MDAI-mediated activation of the Nrf-2 pathway was accompanied by a significant recovery of mitochondrial membrane potential in RAW264.7 cells, confirming the alleviation of oxidative stress at the mitochondrial level. This restoration of mitochondrial health further contributes to breaking the inflammatory cycle and promotes macrophage polarization toward the anti-inflammatory M2 phenotype [48, 49]. In support of this, we observed that MDAI-treated RAW264.7 cells displayed an increased proportion of anti-inflammatory M2 macrophages, accompanied by suppressed NF-κB signaling and downregulated M1 markers. Transcriptomic analysis using gene set enrichment analysis further validated the inhibition of TNF and NF-κB signaling pathways. Functionally, these repolarized M2 macrophages attenuated osteoclastogenesis, as indicated by a reduction in TRAP^+^ cell numbers (Figure 9B). In turn, M2 macrophages secrete a range of anti-inflammatory cytokines and pro-osteogenic factors such as BMP-2 and TGF-β, which collectively establish a regenerative microenvironment conducive to osteoblast differentiation and bone formation [50]. Accordingly, we detected enhanced ALP and ARS staining in BMSCs cultured with conditioned medium from MDAI-primed macrophages, further confirming the functional benefit of macrophage repolarization on osteogenic activity. In summary, this study establishes a mechanistic framework in which ROS-driven inflammation sustains periodontal pathology, while the MDAI-loaded hydrogel reinstates immune homeostasis through Nrf-2/HO-1 activation, thereby suppressing inflammation, inhibiting osteoclast activity and enhancing osteogenesis.

The immune-osteogenic coupling elicited by our system translates into pronounced functional recovery. Micro-CT analysis demonstrated a 76% reduction in the CEJ–ABC distance and restoration of BV/TV to 78.35% (compared with 33.94% in controls), directly correlating with enhanced M2 macrophage infiltration. This multifunctional platform effectively addresses persistent challenges in periodontitis therapy, including limited local drug retention, unidimensional treatment approaches and unresolved immune dysregulation. Beyond periodontitis, the “ROS clearance–immune remodeling–regeneration” paradigm offers a versatile therapeutic framework for other ROS-driven chronic disorders, including diabetic ulcers and rheumatoid arthritis, through adaptable immunomodulatory interventions.

However, several avenues remain for future investigation. Specifically, the effect of this system on pathogenic bacterial populations within periodontal pockets was not evaluated and warrants further study. Additionally, validation of therapeutic efficacy in larger animal models is necessary before clinical translation. Mechanistically, while both 4OI and MDA demonstrated protective effects, they may act through distinct mechanisms, a possibility that is worthy of future investigation.

## Conclusion

In summary, we developed a thermosensitive hydrogel capable of *in situ* polymerization within murine periodontal tissues to enable controlled release of MDAI. The therapeutic effects of MDAI were mediated through two complementary mechanisms: MDA efficiently scavenged ROS, while the released 4OI activated the Nrf-2/HO-1 signaling pathway, amplifying antioxidant responses. This chemico-biological cascade effectively reduced ROS levels and mitigated mitochondrial oxidative damage, resulting in macrophage polarization toward the anti-inflammatory M2 phenotype and suppression of pro-inflammatory cytokine expression. Transcriptomic analyses further confirmed downregulation of inflammatory mediators and inhibition of associated signaling pathways. In a murine model of periodontitis, MDAI released from the hydrogel significantly attenuated local inflammation, promoted M2 macrophage polarization and enhanced alveolar bone regeneration. Collectively, these findings demonstrate a novel, integrated therapeutic strategy for periodontitis management, highlighting the potential of spatiotemporally coordinated antioxidant-immunomodulatory interventions for clinical translation.

## Funding

This work was financially supported by National Natural Science Foundation of China (No. 82401093), Health Profession Clinical Research Funds of Shanghai Municipal Health Commission (No. 20214Y0356), Talent Development Plan funded by Shanghai Fifth People’s Hospital, Fudan University (No. 2024WYRCJY05) and High-level Professional Physician Training Program of Minhang District (2024MZYS14).

*Conflicts of interest statement*. The authors declare that they have no conflict of interest.

## Data Availability

The data that support the findings of this study are available from the corresponding author upon reasonable request.
